# Puberty and Inhibin B in 35 Adolescents With Pituitary Stalk Interruption Syndrome

**DOI:** 10.3389/fped.2020.00304

**Published:** 2020-06-11

**Authors:** Victoria Corvest, Pierre Lemaire, Sylvie Brailly-Tabard, Raja Brauner

**Affiliations:** ^1^Fondation Ophtalmologique Adolphe de Rothschild and Université Paris Descartes, Paris, France; ^2^Université Grenoble Alpes, CNRS, Grenoble INP, G-SCOP, Grenoble, France; ^3^Faculté de médecine Paris Sud, Université Paris Saclay and Assistance Publique-Hôpitaux de Paris, Hôpitaux Universitaires Paris Sud, CHU Bicêtre, Service de Génétique Moléculaire, Pharmacogénétique, Hormonologie, Le Kremlin-Bicêtre, France

**Keywords:** cryptorchidism, GnRH, hypogonadotropic hypogonadism, inhibin B, micropenis, pituitary stalk interruption syndrome, pubertal delay

## Abstract

**Background:** In patients with pituitary stalk interruption syndrome (PSIS), long-term follow-up is necessary to address their gonadotrophic status. The objectives of this study were (1) to describe pubertal features of and (2) to assess the ability of serum inhibin B concentration to predict hypogonadotropic hypogonadism (HH) in patients with PSIS.

**Methods:** This retrospective single-center study included 35 patients with PSIS and known gonadotrophic status for whom a serum sample preserved at −22°C (collected at initial evaluation or later) was available for measuring inhibin B by the same hormonal immunoassay method.

**Results:** Among the 21 boys, 15 had normal puberty (early in two), and six had partial (*n* = 2) or complete (*n* = 4) HH. Among the 14 girls, five had normal puberty (early in one)—four with regular menses and one in the process of puberty—, four had complete HH, and five had amenorrhea (primary in three and secondary in two) after normal pubertal development, despite a normal pubertal gonadotropin response to gonadotropin-releasing hormone test. These were considered as having partial HH. Only three boys had values over the normal lower range for serum inhibin B concentrations despite partial (*n* = 2) or complete (*n* = 1) HH. Inhibin B concentrations were low in all girls with complete HH, normal in all those with partial HH except in one and in those with normal puberty except in two. Considering boys and girls together, the occurrence of under-range inhibin B was significantly higher in those with HH than in those without (47 vs. 10%, *p* = 0.02). All 15 patients with HH had associated thyroid-stimulating hormone and adrenocorticotropic hormone deficiency except for 3 girls with partial HH.

**Conclusions:** Under-range inhibin B concentrations in patients with PSIS might be suggestive of HH. These concentrations provide a simple first-line predictive test, especially in boys.

## Introduction

Pituitary stalk interruption syndrome (PSIS) is a congenital anomaly of the pituitary gland characterized by a combination of specific findings on magnetic resonance imaging (MRI), including an interrupted pituitary stalk, an absent or ectopic posterior pituitary gland and anterior pituitary hypoplasia or aplasia (1). PSIS can also be associated with other midline abnormalities and variable endocrine disorders. Because of the heterogeneity in its clinical, biological and imaging presentations, efforts have focused on distinguishing between patients with isolated growth hormone (GH) deficiency and those with multiple hypothalamic-pituitary deficiencies (2). These deficiencies may include thyroid-stimulating hormone (TSH), adrenocorticotropic hormone (ACTH), gonadotropin [luteinizing hormone (LH) and follicle-stimulating hormone (FSH)] and rarely antidiuretic hormone deficiencies. In patients with PSIS, long-term follow-up is necessary to address their gonadotrophic status once they have reached pubertal age.

Thus, the diagnosis of hypogonadotropic hypogonadism (HH) is difficult, especially during the prepubertal period, during which HH is generally not clinically apparent, and the functional assessment of the hypothalamic-pituitary-gonadal axis is limited due to its physiological quiescence at this age. Making an early diagnosis in children before they present with pubertal delay offers the opportunity to initiate hormone replacement therapy at the normal age of pubertal onset. This could optimize growth, bone mineralization, and psychological well-being and eventually help to anticipate the management of future fertility. The transient postnatal activation of the gonadotrophic axis, called the minipuberty of early infancy and consisting of a surge in gonadotropins and gonadal steroids (3–6), provides a window of opportunity (from birth to approximately 6 months of age in boys and to 2 years of age in girls) to diagnose HH.

Different biological markers, including basal measurements and stimulation tests, have been proposed as early discriminators between HH and constitutional delay of puberty; these markers include basal plasma concentrations of testosterone, LH and FSH, gonadotropin-releasing hormone (GnRH) tests, or stimulation tests with human chorionic gonadotropin. However, they appear to be less informative about the effective function of the hypothalamic-pituitary-gonadal axis and have limited discriminatory ability because the concentrations are normally low or undetectable during childhood and show significant variability and overlap in the responses at pubertal age (7, 8).

The advent of new markers of gonadal function, such as serum inhibin B or anti-Müllerian hormone concentration, may offer simple alternatives to dynamic testing measurements in HH (9, 10). Inhibin B is a glycoprotein hormone produced by Sertoli cells in the testis or by granulosa cells in the ovary and is stimulated by and involved in feedback inhibition of FSH (11–16). Inhibin B reflects testicular and ovarian function and could be used as an interesting tool because its concentrations are detectable during prepuberty and show significant changes during normal puberty (11, 17, 18). Few reports are available on the potential use of serum inhibin B for diagnosing HH during childhood, especially at an early age, for girls (19) or in cases of combined pituitary hormone deficiency, such as in PSIS (9, 20–22).

The objectives of this study were (1) to describe the pubertal features of and (2) to assess the ability of serum inhibin B concentration to predict HH in patients with PSIS.

## Materials and Methods

### Setting

In this retrospective single-center study, all patients monitored for hypothalamic-pituitary deficiency with PSIS by a single senior pediatric endocrinologist (R. Brauner) in a university hospital between 1978 and 2019 were eligible. We included the patients with available serum sample preserved at −22°C (collected at initial evaluation or later, at various ages) for measuring inhibin B by the same hormonal immunoassay method, as well as with known gonadotrophic status.

### Participants

**Figure 1:**
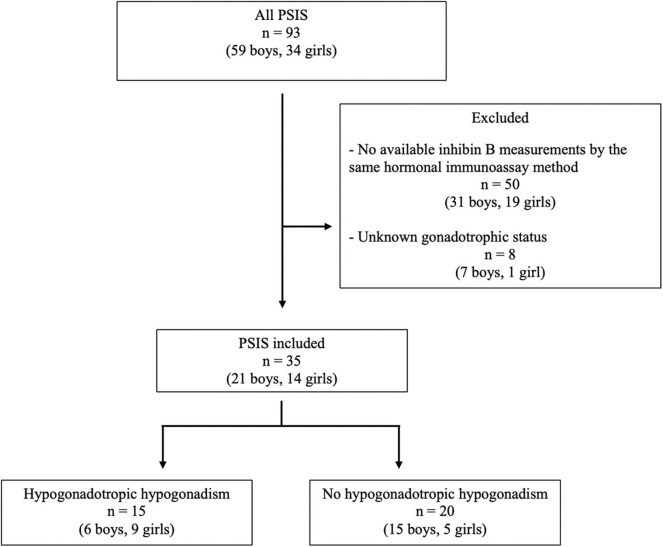
Flowchart of the inclusion of the patients with PSIS.

Thirty-five patients (21 boys, 14 girls) from a total of 93 (38%) patients were included (Figure 1). Five of the 21 boys (patients 4, 6, 8, 14, and 20) had been included in our previous evaluation of inhibin B in HH (9). The characteristics of the 35 included patients were similar to those of the 58 excluded patients, except for the age at last evaluation, as the included patients were significantly older (19.1 ± 5.0 years and 13.0 ± 5.2 years, respectively, *p* < 0.01). All patients had PSIS with GH deficiency, with a peak GH response of <20 mU/L or 6.7 ng/mL after two pharmacological stimulation tests.

### Methods

The following data were collected at the initial clinical evaluation: sex, age at diagnosis, perinatal history including gestational age, delivery by breech and/or cesarean section, acute perinatal distress (Apgar score <7 at 5 min and/or neonatal resuscitation), aspect of the pituitary on MRI including the height of the anterior pituitary (1), presenting with symptoms, antecedence of hypoglycaemia, associated malformations (including ophthalmic), and micropenis or cryptorchidism in boys. Micropenis was defined as a penis length of <2 SD below the lower normal range (23). Age and pubertal status, including clinical evaluation of testicular volume of the boys, were also collected at the last evaluation.

The initial biological evaluation included the peak GH concentration after a stimulation test (ornithine, arginine-insulin and/or glucagon test), the plasma concentrations of insulin-like growth factor 1 (IGF1), cortisol at 0800 h, ACTH, TSH, and free thyroxin. As various GH assays were used over the study period, we expressed the peak GH concentration in mU/L using conversion factors. Pituitary functions other than GH (ACTH and TSH) were evaluated at the time of diagnosis and during follow-up if normal to enable the diagnosis of delayed deficiencies. ACTH deficiency was diagnosed by plasma basal cortisol concentrations below 40 μg/L in neonates and below 80 μg/L in older children, with no increase during hypoglycaemia; ACTH test was not performed. TSH deficiency was diagnosed by a plasma free thyroxin concentration below 12 pmol/L with low or normal TSH concentration (2).

Pubertal Tanner stages (24, 25) were clinically evaluated at regular follow-up visits by the same physician (R. Brauner). All patients, but two girls (cases 34 and 35), were in prepubertal age at diagnosis. In boys, puberty was considered to be absent (complete HH) when testicular volume was below 4 mL at 14 years without subsequent increase (26, 27). Puberty was also considered abnormal (partial HH) in two boys with a slight initial increase in their testicular volume (9 mL in case 4 and 6 mL in case 7) but no further development, and with plasma testosterone concentrations remaining undetectable. In girls, the diagnosis of HH was made when breast development was absent at 13 years without subsequent increase. Primary or secondary amenorrhea after complete breast development, despite a normal pubertal gonadotropin response to GnRH test, was considered as secondary to partial HH as there is no alternative diagnosis. Early puberty was defined as the onset of pubertal development between 8 and 10 years in girls and between 10 and 11 years in boys (28, 29). Plasma prolactin concentrations were measured in all patients except two (cases 1 and 9) and were normal.

No patient in this series was exposed to gonadotropins nor to any exogenous gonadal hormonal replacement treatment before biological evaluation. A GnRH test was performed at 14.3 ± 4.1 years (except in six boys and three girls with normal pubertal development) in addition to measurements of basal plasma testosterone in boys or estradiol concentrations in girls. For the GnRH stimulation test, we used Gonadorelin (Relefact, Ferring SAS, 100 μg/m2, maximum 100 μg), with serum samples collected at 0, 30, 60, and 90 min after the injection. LH and FSH concentrations were measured using a two-site monoclonal immunoradiometric assay (LH-Coatria and FSH-Coatria; bioMerieux, SA, Marcy-l'Etoile, France). The within-assay coefficients of variation (CV) ranged from 3 to 7% for LH and 4.5% for FSH. The between-assay was 11.4 and 7.8% respectively (30). Testosterone was extracted with di-ether and measured using a radioimmunoassay (TESTO-CT2 Cis Bio International, Gif sur Yvette, France). Estradiol was extracted with ether and measured using a radioimmunoassay (Estradiol-2; Sorin Biomedica, Antony, France). The within- and between-assay CV were respectively 4.5 and 5% for testosterone, and 4 and 7% for estradiol. Serum LH, FSH, testosterone and estradiol concentrations were measured using various radioimmunoassays during the study period. Each new assay for a given hormone was cross-correlated with the previous method to ensure comparable results for a given parameter throughout the study period. Serum inhibin B concentrations were measured using the same enzyme immunometric assay in all patients (Ansh Labs reagents, Webster, TX, US) (31) to ensure comparable results among patients and throughout the whole study period. The lower limit of detection was 3 pg/mL.

### Statistical Analysis

We statistically compared inhibin B concentrations and other different variables of interest among the patients of each sex with (*n* = 15, six boys, nine girls) and without (*n* = 20, 15 boys, five girls) HH. Inasmuch as inhibin B concentrations vary greatly from birth to adulthood, they were also graphically presented considering the different age and pubertal classes of the patients (14, 17, 31, 32). The values found in the patients were analyzed according to the ranges reported. The under-range inhibin B concentrations correspond to values below these ranges.

Qualitative results are presented as percentages, and quantitative results are presented as the means and standard deviations and expressed as mean ± SD. Between-group comparisons were performed using Fisher's exact test for qualitative variables and the non-parametric Mann–Whitney *U*-test for quantitative variables. An investigation of the distribution, means and medians and application of the Shapiro-Wilk normality test confirmed that groups were not normally distributed. A *p*-value < 0.05 was considered significant. Receiver operating characteristic (ROC) curves were used to evaluate the diagnostic performance of baseline inhibin B in predicting HH and determine the optimal cut-off values. The area under the ROC curve (AUC) was expressed as means. Descriptive and analytical statistics were conducted with RStudio software.

## Results

### Patient Characteristics

No intrauterine growth retardation or post-term birth (>41 weeks of amenorrhea) was observed in the medical charts; two patients (cases 4 and 6) were born preterm (before 37 weeks). Breech presentation at birth occurred in 21% of patients; cesarean section was the method of delivery in 31% of patients, and acute perinatal distress occurred in 17% of patients (Table 1). A total of 36% of patients presented with one or multiple hypoglycaemic episodes. Isolated GH deficiency was identified in 43% of patients and was associated with ACTH deficiency, TSH deficiency, and/or HH in 37, 57, and 43% of patients, respectively. The posterior pituitary gland was ectopic, absent or normal in 29 (83%), three (9%) and three (9%) patients, respectively. The pituitary stalk was interrupted, absent, thin or normal in 16 (46%), 11 (31%), three (9%), and five (14%) patients, respectively. All patients had abnormal posterior pituitary glands and/or abnormal pituitary stalks.

**Table 1:** Characteristics of the 35 adolescents with PSIS.

		***n* (%)**	**mean ± SD**
Sex	Boys	21 (60)	
	Girls	14 (40)	
Age at diagnosis, years			5.3 ± 5.2
Age at last evaluation, years			19.1 ± 5.0
Breech presentation		7 (21)	
Perinatal distress		5 (17)	
Hypoglycemia		12 (36)	
Micropenis (boys)		5 (24)	
Cryptorchidism (boys)		4 (20)	
GH peak, mU/L			5.0 ± 3.7
IGF1, nmol/L			3.3 ± 2.1
Cortisol, nmol/L			305.1 ± 256.6
Free thyroxin, pmol/L			12.4 ± 4.6
Isolated GH deficiency		15 (43)	
ACTH deficiency		13 (37)	
TSH deficiency		20 (57)	
Gonadotropin deficiency		15 (43)	

*Data are available in all except in breech presentation [available in (33)], perinatal distress (29), hypoglycemia (34), cryptorchidism (20), and IGF1 (34). Conversion factors to SI units: IGF1 1 ng/mL = 0.13 nmol/L; cortisol 1 ng/mL = 2.76 nmol/L*.

### Puberty in Boys

Among the 21 boys, six had absent or incomplete pubertal development with partial (*n* = 2) or complete (*n* = 4) HH, respectively, two (patients 3 and 10) had central early puberty, and 13 experienced normal puberty (Table 2). The two boys with partial HH (patients 4 and 7) had a slight increase in their testicular volume but no progression with plasma testosterone concentration remaining undetectable. Of the five boys with a history of micropenis, three had HH. In the remaining two (with penis length at 17 and 25 mm), patient 3 experienced early puberty, and the MRI showed olfactory bulb aplasia, while patient 13 had cleft lip and palate. Both developed normal clinical and biological puberty. Of the four boys with a history of cryptorchidism (unilateral in two), three had HH and one (patient 19) had normal pubertal development without HH but with anorectal malformation. Cryptorchidism was treated by surgery in cases 4, 15, and 19, but no precision was found in case 7.

**Table 2:** Individual data of the boys with PSIS.

**Case**	**Age at diagnosis years**	**MRI**			**Ophthalmic malformations or anomalies**	**Other malformations or anomalies**	**Genital anomalies**	**Puberty**	**GnRH test**			**Testosterone nmol/L**	**Age at inhibin B years**	**Inhibin B ng/L**	**HP deficiencies**	**Last evaluation**	
		**Posterior pituitary**	**Pituitary stalk**	**Anterior pituitary height mm**					**Age years**	**LH peak IU/L**	**FSH peak IU/L**					**Age years**	**TV mL**
1	0.1	Ectopic	Absent	NA	Strabismus, astigmatism, hyperopia	No	a	Absent	12.0	0.2	0.6	<0.24	12.0	11	GH, ACTH, TSH, LH/FSH	13.0	2
2	0.1	Ectopic	Absent	2.5	No	No	a	Absent	12.6	<0.1	0.5	<0.24	12.6	<16.6	GH, ACTH, TSH, LH/FSH	14.0	2
3	0.9	Ectopic	Absent	NA	Bilateral nerve atrophy	Olfactory bulbs aplasia	a	Early puberty	17.5	23.0	10.4	26.21	17.5	168	GH, ACTH, TSH	19.4	10
4	1.3	Ectopic	Interrupted	0.5	Ptosis	Arnold Chiari syndrome	b	Partial	14.8	3.0	4.2	<0.24	1.3	142	GH, ACTH, TSH, LH/FSH (partial)	26.8	9
5	2.7	Ectopic	Normal	4	No	No	No	Normal	12.4	8.2	4.2	1.14	12.4	125	GH, TSH	14.7	15
6	2.7	Ectopic	Absent	0.5	No	Cerebellar atrophy	a	Absent	13.8	0.9	2.2	0.24	2.7	281	GH, ACTH, TSH, LH/FSH	29.3	2
7	2.8	Ectopic	Interrupted	2	No	Diabetes mellitus	b	Partial	15.1	3.7	8.4	0.21	2.8	94	GH, ACTH, TSH, LH/FSH (partial)	15.1	6
8	3.1	Ectopic	Interrupted	2	No	No	No	Normal	3.1	3.2	4.9	0.04	3.1	98	GH	16.3	15
9	3.4	Normal	Absent	3	No	Temporal arachnoid cyst	No	Normal	14.4	10.3	6.3	15.60	3.4	122	GH	19.1	15
10	3.5	Ectopic	Thin	2	No	Pharyngeal malformation	No	Early puberty	11.7	16	3.2	13.52	3.6	74	GH	13.5	Adult
11	3.6	Ectopic	Interrupted	2	No	No	No	Normal	14.9	20.6	7.4	17.13	3.6	85	GH	14.9	15
12	4.0	Ectopic	Thin	NA	No	No	No	Normal	14.5	6.4	3.7	0.49	4.0	395	GH, TSH	17.6	15
13	4.8	Ectopic	Absent	0	No	Cleft lip and palate	a	Normal	17.0	10.4	3.4	20.08	17.0	206	GH	17.0	15
14	4.9	Ectopic	Absent	1	No	Arnold Chiari syndrome	NA^*^	Normal	NA	NA	NA	23.23	5.1	142	GH	15.6	15
15	5.1	Absent	Interrupted	NA	Astigmatism, hyperopia	No	b	Absent	13.4	0.8	0.7	<0.24	5.1	16	GH, ACTH, TSH, LH/FSH	15.4	2
16	5.5	Ectopic	Normal	3	No	Hemophilia	No	Normal	NA	NA	NA	NA	5.5	81	GH	18.7	15
17	5.6	Ectopic	Thin	4.3	No	No	No	Normal	NA	NA	NA	5.27	5.6	124	GH, TSH, ACTH (partial)	17.5	15
18	6.4	Ectopic	Thin	4	No	No	No	Normal	NA	NA	NA	NA	6.4	29	GH	15.1	15
19	6.7	Ectopic	Normal	NA	No	Anorectal malformation	b	Normal	17.8	37.2	31.2	21.81	6.5	109	GH	17.8	13
20	7.0	Ectopic	Interrupted	4	No	No	No	Normal	NA	NA	NA	22.54 (18.4 yr)	10.4	315	GH	18.4	15
21	10.4	Ectopic	Absent	0	No	No	No	Normal	NA	NA	NA	NA	10.4	158	GH, TSH	14.8	12

*NA, not available; a, micropenis; b, cryptorchidism; TV, testicular volume*.

* *No micropenis, localization of the testes NA*.

*Conversion factors to SI units: testosterone 1 ng/mL = 3.47 nmol/L; inhibin B 1 pg/mL = 1 ng/L*.

All six boys with HH had associated ACTH and TSH deficiencies. These six boys had significantly lower ages at diagnosis, basal plasma IGF1, cortisol, free thyroxin, and testosterone concentrations, peak LH after the GnRH test, and testicular volume at the last evaluation than the other 15 boys, while their peak FSH after the GnRH test and their ages at the GnRH test, inhibin B and final evaluations were similar (Table 3).

**Table 3:** Comparison between boys with and without hypogonadotropic hypogonadism (HH) in PSIS.

	**With HH (*n* = 6)**	**Without HH (*n* = 15)**	***p***
Age at diagnosis, years	2.0 ± 1.9	4.8 ± 2.3	**0.01**
Age at last evaluation, years	18.9 ± 7.2	16.7 ± 1.9	0.79
Micropenis, *n* (%)	3 (50)	2 (13)	0.12
Cryptorchidism n/total (%)	3/6 (50)	1/14 (7)	0.06
Anterior pituitary height, mm	1.4 ± 1.0	2.4 ± 1.5	0.27
GH peak, mU/L	6.8 ± 4.7	4.7 ± 3.7	0.24
IGF1, nmol/L	1.6 ± 1.4	3.7 ± 1.9	**0.03**
Cortisol, nmol/L	61.0 ± 57.7	393.9 ± 304.8	**<0.01**
Free thyroxin, pmol/L	7.8 ± 2.3	14.8 ± 4.7	**<0.01**
Age at GnRH testing, years	13.6 ± 1.2	13.7 ± 4.5	0.61
LH peak, IU/L	1.4 ± 1.5	15.0 ± 10.6	**<0.01**
FSH peak, IU/L	2.8 ± 3.1	8.3 ± 8.9	0.06
Testosterone, nmol/L	0.1 ± 0.1	13.9 ± 9.7	**<0.01**
TV, mL	3.8 ± 3.0	14.3 ± 1.5	**<0.01**
Age at inhibin B, years	6.1 ± 5.0	7.6 ± 4.8	0.27
Inhibin B, ng/L	90.6 ± 108.7	148.7 ± 95.1	0.16
Underrange inhibin B, n (%)	3 (50)	0 (0)	**0.02**

*Mean ± SD, TV, testicular volume. Conversion factors to SI units: IGF1 1 ng/mL = 0.13 nmol/L; cortisol 1 ng/mL = 2.76 nmol/L; testosterone 1 ng/mL = 3.47 nmol/L; inhibin B 1 pg/mL = 1 ng/L*.

*Bold values are statistically significant p-value*.

**Figure 2:**
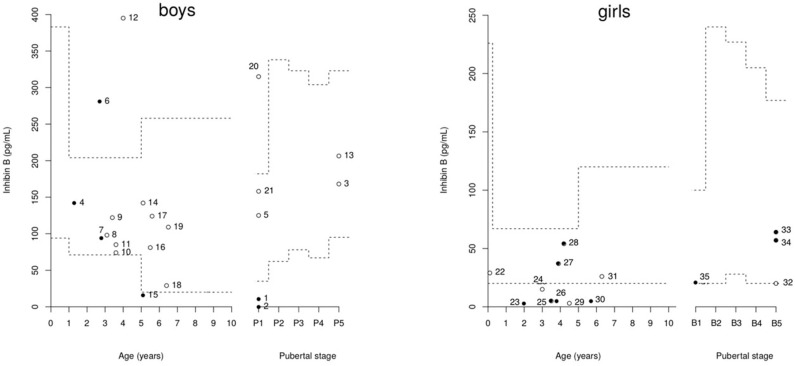
Distribution of inhibin B concentrations in 21 boys and 14 girls with PSIS.Broken lines represent the reference ranges according to age and pubertal stage (14, 17, 31, 32). The closed circles represent patients with hypogonadotropic hypogonadism (HH), and the open circles represent those without HH. Cases 4, 7, 25, 27, 28, 33, and 34 have partial HH.

The individual inhibin B values are plotted in Figure 2. Only three boys had values over the normal lower range for serum inhibin B concentrations despite partial (patients 4 and 7) or complete (patient 6) HH.

**Figure 3:**
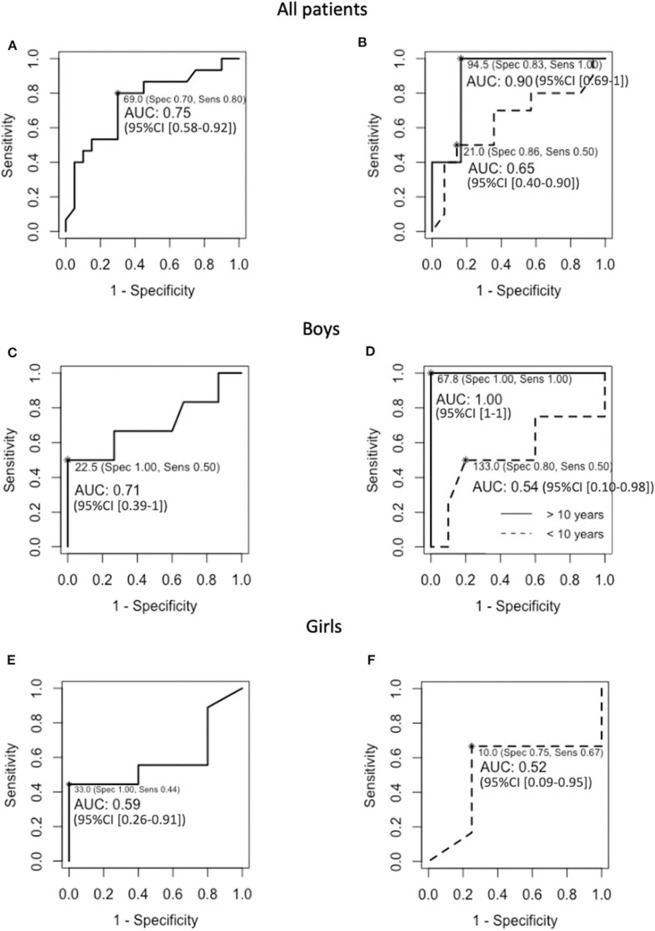
ROC curves for baseline inhibin B in **(A)** the 35 included PSIS patients, **(B)** the 24 and 11 PSIS patients that were younger or older than 10 years of age at inhibin B evaluation, respectively, **(C)** the 21 PSIS boys, **(D)** the 14 and seven PSIS boys that were younger or older than 10 years of age at inhibin B evaluation, respectively, **(E)** the 14 PSIS girls, and **(F)** the 10 PSIS girls that were younger than 10 years of age at inhibin B evaluation. NB, The number of girls that were older than 10 years of age at inhibin B evaluation (*n* = 4) was insufficient to estimate the diagnostic performance and to calculate the 95% CI of the AUC.

The mean inhibin B concentrations were similar in the boys with or without HH, as well as the mean age at inhibin B measurement (Table 3). However, the occurrence of under-range inhibin B concentration was significantly higher in patients with HH than in those without HH. The sensitivity of under-range inhibin B concentration to detect HH was 50% (95% CI [12–88%]), and its specificity was 100% (95% CI [78–100%]). The number of HH and non-HH patients included in the analysis is 6 and 15 boys, respectively (Table 3). The cut-off values of inhibin B that yielded maximum sensitivity and specificity were 23 pg/mL for the whole group of boys [50 and 100%, respectively, AUC 0.71 (95% CI [0.39–1])] and 68 pg/mL in boys older than 10 years of age (100% for both sensitivity and specificity, AUC 1 [95% CI (1)]) (Figure 3).

### Puberty in Girls

Among the 14 girls, four had absent pubertal development (complete HH), and five had complete pubertal development but primary (three girls) or secondary (two girls) amenorrhea (partial HH) (Table 4). Among these, the gonadotropin response to the GnRH test was pubertal normal (patients 27, 28, and 34) or high (patient 25); in patient 33, whose levels were not evaluated by the GnRH test, the basal concentrations at 27 years were LH 6.1 U/L, FSH 9.8 U/L and estradiol 28 pg/mL. Among the five other girls, one had early and rapid puberty followed by regular menstruations at the final evaluation at 25.8 years (patient 29), one was in the process of puberty (patient 22), and three menstruated regularly at 20.4 years (patient 24), 12.8 years (patient 31), and 26.9 years (patient 32).

**Table 4:** Individual data of the girls with PSIS.

**Case**	**Age at diagnosis years**	**MRI**			**Ophthalmic malformations or anomalies**	**Other malformations or anomalies**	Puberty^*^	**GnRH test**			**Estradio pmol/L**	**Age at inhibin B years**	**Inhibin B ng/L**	**HP deficiencies**	**Age at last evaluation years**
		**Posterior pituitary**	**Pituitary stalk**	**Anterior pituitary height mm**				**Age years**	**LH peak IU/L**	**FSH peak IU/L**					
22	0.1	Ectopic	Interrupted	NA	Strabismus	Septal agenesis, cerebral ventriculomegaly	Normal (in progress): B4P4M0	10.7	5.9	7.8	<58.7	0.1	29	GH, TSH	13.0
23	2.0	Ectopic	Interrupted	2	Strabismus, unilateral amblyopia	No	Absent: B1P4M0	15.8	1.6	0.6	36.7	2.0	3	GH, TSH, ACTH (transient), LH/FSH	25.9
24	3.0	Ectopic	Absent	2	No	No	Normal: B5P5M1	NA	NA	NA	124.8	3.0	15	GH	20.4
25	3.5	Ectopic	Normal	4	No	No	Partial: B5P5, SA	14.0	85.0	20.0	143.2	3.5	5	GH, LH/FSH (partial)	23.8
26	3.8	Ectopic	Interrupted	1	No	Absent sella turcica	Absent: B1P2M0	11.6	0.4	<0.2	25.7	3.8	5	GH, ACTH, TSH, LH/FSH	22.3
27	3.9	Ectopic	Interrupted	1	Strabismus	No	Partial: B5P5M0, PA	15.8	37.0	21.0	47.7	3.9	37	GH, TSH, LH/FSH (partial)	20.8
28	4.2	Ectopic	Interrupted	2	Optic nerve hypoplasia	No	Partial: B5P4, PA	14.2	17.0	12.0	44.1	4.2	54	GH, ACTH, TSH, LH/FSH (partial)	19.9
29	4.5	Normal	Interrupted	4	No	No	Early: B5P5M1	14.8	37.0	21.0	110.1	4.5	3	GH	25.8
30	5.7	Ectopic	Absent	NA	Strabismus, unilateral amblyopia	No	Absent: B1P3M0	11.5	<0.4	<0.4	NA	5.7	5	GH, ACTH, TSH, LH/FSH	14.1
31	6.3	Absent	Interrupted	2	Strabismus	Leucomalacia	Normal: B5P5M1	11.8	17.0	12.0	62.4	6.3	26	GH, TSH	12.8
32	7.8	Absent	Normal	5	No	No	Normal: B5P5M1	NA	NA	NA	NA	26.9	20	GH	26.9
33	9.0	ectopic	Interrupted	4	No	No	Partial: B5P5, SA	NA	NA	NA	135.8	14.1	64	GH, LH/FSH (partial)	27.0
34	18.5	Normal	Interrupted	4	Optic nerve atrophia, congenital glaucoma	De Morsier syndrome	Partial: B5P3M0, PA	18.5	21.0	11.0	84.4	18.5	57	GH, ACTH, TSH, LH/FSH (partial)	22.8
35	27.8	Ectopic	Absent	1	No	No	M0 (B: NA, P: NA)	27.8	<1	<1	NA	27.8	21	GH, ACTH, TSH, LH/FSH	27.8

*NA, not available*.

* *BPM spontaneous puberty stages according to Marshall and Tanner (24), SA, secondary amenorrhea; PA, primary amenorrhea*.

*Case 33: basal LH 6.1 IU/L, basal FSH 9.8 IU/L, estradiol 28 pg/mL at last evaluation at 27 years old*.

*Conversion factors to SI units: estradiol 1 pg/mL = 3.67 pmol/L; inhibin B 1 pg/mL = 1 ng/L*.

All nine girls with HH had associated ACTH and TSH deficiencies except for three girls with partial HH, while ACTH deficiency was transient in one (patient 23). These nine girls had significantly lower free thyroxin concentration than the other five girls, while their basal serum IGF1, cortisol, and estradiol concentrations, peak LH and FSH after the GnRH test, and ages at diagnosis, GnRH test, inhibin B and final evaluations were similar (Table 5).

**Table 5:** Comparison between girls with and without hypogonadotropic hypogonadism (HH) in PSIS.

	**With HH (*n* = 9)**	**Without HH (*n* = 5)**	***p***
Age at diagnosis, years	8.7 ± 8.7	4.3 ± 3.0	0.61
Age at last evaluation, years	22.7 ± 4.2	19.8 ± 6.8	0.36
Anterior pituitary height, mm	2.4 ± 1.4	3.3 ± 1.5	0.29
GH peak, mU/L	4.2 ± 3.1	5.2 ± 3.7	0.64
IGF1, nmol/L	3.7 ± 1.7	4.0 ± 3.6	0.57
Cortisol, nmol/L	317.5 ± 230.4	308.4 ± 69.0	0.80
Free thyroxin, pmol/L	10.0 ± 3.1	14.8 ± 2.5	**0.02**
Age at GnRH testing, years	16.2 ± 5.2	12.5 ± 2.1	0.28
LH peak, IU/L	20.3 ± 29.4	20.0 ± 15.8	0.61
FSH peak, IU/L	8.1 ± 9.1	13.6 ± 6.7	0.35
Estradiol, pmol/L	73.8 ± 48.5	74.5 ± 56.2	1
Age at inhibin B, years	9.3 ± 8.9	8.2 ± 10.7	0.80
Inhibin B, ng/L	27.9 ± 25.4	18.6 ± 10.3	0.64
Under-range inhibin B, *n* (%)	4 (44)	2 (40)	1

*Mean ± SD. Conversion factors to SI units: IGF1 1 ng/mL = 0.13 nmol/L; cortisol 1 ng/mL = 2.76 nmol/L; estradiol 1 pg/mL = 3.67 pmol/L; inhibin B 1 pg/mL = 1 ng/L*.

*Bold values are statistically significant p-value*.

The individual inhibin B values are plotted in Figure 2. The serum inhibin B concentrations were low in all four girls with complete HH (on the lower limit for patient 35) and normal in all those with partial HH except in patient 25. These concentrations were normal in those with normal puberty except in patients 24 and 29.

The mean inhibin B concentrations and the occurrence of under-range inhibin B concentration were similar in the girls with or without HH as well as the mean age at inhibin B measurement (Table 5). The sensitivity of under-range inhibin B concentration to detect HH was 44% (95% CI [14–79%]), and its specificity was 60% (95% CI [15–95%]). The number of HH and non-HH patients included in the analysis is 9 and 5 girls, respectively (Table 5). The cut-off value of inhibin B that yielded the maximum sensitivity and specificity was 33 pg/mL for the whole group of girls [44 and 100%, respectively, AUC 0.59 (95% CI [0.26–0.91])]. This value was 10 pg/mL in young girls (67% and 75% respectively, AUC 0.52 (95% CI [0.09–0.95])] (Figure 3). The number of girls that were older than 10 years of age at inhibin B evaluation (*n* = 4) was insufficient to estimate the diagnostic performance and to calculate the 95% CI of the AUC.

Considering boys and girls together, the occurrence of under-range inhibin B concentration was significantly higher in those with HH than in those without (47 vs. 10%, *p* = 0.02).

## Discussion

The objectives of this study were (1) to describe pubertal features of and (2) to assess the ability of serum inhibin B concentration to predict HH in patients with PSIS. This study shows the heterogeneity of pubertal abnormalities in these patients. Among the 21 boys, 15 had normal puberty (early in two), and six had partial (*n* = 2) or complete (*n* = 4) LH/FSH deficiencies. Among the 14 girls, five had normal puberty (early in one)—four with regular menses and one in the process of puberty—, four had complete LH/FSH deficiencies, and five had amenorrhea (primary in three and secondary in two) after normal pubertal development, despite a pubertal increase in LH/FSH after the GnRH test in the four girls evaluated. These were considered as having partial HH. Only three boys, including the two with partial HH, had inhibin B values over the normal lower range despite having HH. Inhibin B concentrations were low in all girls with complete HH, normal in all those with partial HH except in one and in those with normal puberty except in two. All 15 patients with HH had associated TSH and ACTH deficiency except for 3 girls with partial HH. The boys with HH had significantly lower IGF1 concentrations at diagnosis than those without HH, despite similar GH peak. Their significantly lower free thyroxin concentrations possibly explain this difference.

### Boys

Similar to the results from other authors, we found that a history of micropenis and/or cryptorchidism, testis dimensions that do not increase by pubertal age and no LH increase after the GnRH test are highly suggestive of HH, but individually, they are not discriminative for diagnosis (33, 34). Thus, two patients had micropenis followed by normal pubertal development. One could hypothesize that it could be linked to intra-uterine GH deficiency. The only patient with cryptorchidism followed by normal puberty had anorectal malformation. Braslavsky et al. reported on 21 boys evaluated before 6 months of age with congenital multiple hypopituitarism and anatomical abnormalities of the hypothalamic-pituitary region on MRI, among whom 14 had HH (22). The authors found a significant association between abnormal genitalia and abnormally low concentrations of LH and testosterone. Abnormal genitalia had a 93% positive predictive value, 100% sensitivity and 85% specificity for HH, whereas the existence of normal external genitalia excluded HH in all cases.

The present study shows that an under-range single measurement of inhibin B concentration might be suggestive of HH in boys with PSIS. However, three boys had normal serum inhibin B concentrations despite partial or complete HH. Two of them had been included in our previous study, which used another method to measure inhibin B (Oxford Bio-Innovation reagents, Serotec, Oxford, UK) and also showed normal concentrations [see patients in (9)]. The association with micropenis in one and cryptorchidism in the two others suggests that HH was present before birth. Braslavsky et al. reported that infants with HH had lower inhibin B concentrations; nevertheless, inhibin B had no diagnostic significance for HH, as only 58% of the boys with HH had inhibin B concentrations below the normal range (22). Conversely, Rottembourg et al. found low inhibin B (31–114 pg/mL) concentrations in four boys with PSIS and spontaneous puberty, one of whom had a history of micropenis and cryptorchidism; the authors suggested that the boys had gonadotropin dysfunction that was not detectable by conventional GnRH testing (20). Coutant et al. evaluated 15 boys with congenital HH combined with pituitary hormone deficiency, among whom 11 had stalk agenesis (21). In boys at genital stage two, for an inhibin B concentration no >65 pg/mL, the true positive rate for diagnosing HH was 87%, whereas the false negative rate was 20%, and no combination or ratio of hormones achieved a similar performance. We found a specificity and sensitivity of 100% for a lower limit of 68 pg/mL in boys older than 10 years of age (mostly at prepubertal stage). The authors used a method to obtain measurements of the concentration of inhibin B whose correlation coefficient with our results is 0.96. This good diagnostic performance of inhibin B at pubertal age may be considered with caution given the small sample size, setting a trend that requires further study to assess more accurate threshold values for clinical use. A recent review of Grispon et al. confirmed that anti-Müllerian hormone and inhibin B concentrations stand out as the most useful biomarkers for the diagnosis of hypogonadism during childhood (35).

### Girls

The occurrence of primary or secondary amenorrhea without LH/FSH deficiency was also reported in four girls with PSIS (20). The median peaks were 30 U/L for LH and 11 U/L for FSH. These authors discussed the possibility of subtle disturbances of gonadotropin pulsatility and classified them as partial HH. In this series, only one (with an isolated GH deficiency) of the other eight girls studied underwent normal puberty and had regular menses, and seven had no pubertal development and low peak LH (<0.8 U/L) and FSH (<1.5 U/L) levels.

We found a dissociation of low inhibin B concentration despite normal pubertal development in two girls with samples collected at 3 and 4.5 years. Binder et al. evaluated the accuracy of endocrine testing for detecting HH by comparing nine girls with HH of various etiologies to 12 with constitutional delay of growth and puberty (19). The authors found that the inhibin B concentration (cut-off of 20 pg/mL) was the only basal measurement that distinguished between the two situations, which also corresponded to a lower limit of the inhibin B reference age for healthy girls at Tanner stage two. Regardless of age, we found a poor diagnostic performance of inhibin B, with low sensitivity, specificity and AUC, with mostly prepubertal patients included in our analysis. We found a higher predictive value of inhibin B in males than in girls,. This result is in agreement with the findings of Grinspon et al. that inhibin B is quite low physiologically in prepubertal girls (35), suggesting that inhibin B could be less adequate to assess gonadal function in girls.

### Early Central Puberty

Early central puberty occurred in one girl and two boys, one of whom had bilateral nerve atrophy and olfactory bulb aplasia. This boy will possibly develop partial HH after early puberty. However, we classified him as having no HH because his testicular volume was 10 mL at 19.4 years with LH/FSH increase after the GnRH test and plasma testosterone concentration within the adult normal range. The association between olfactory bulb aplasia and PSIS has been previously reported in one adult (36). The two boys had been treated with a GnRH analog, after which they exhibited normal pubertal development. The occurrence of early puberty in patients with congenital hypothalamic-pituitary deficiency has been previously reported (37, 38), including two patients with septo-optic dysplasia. Due to the dates in which these descriptions were reported, no MRI data were available.

### Strengths and Limitations of the Study

A strength of our study is that we performed a homogeneous and comparable evaluation of all patients. The evaluation criteria were all objective (quantitative). Furthermore, this cohort of PSIS patients, although small, represents one of the largest reported to date to our knowledge.

This study also had some limitations. This was a retrospective study with a poor sample size from which it was not possible to detect all the significant differences among groups, although the use of a single investigator limited the inherent bias. The exclusion of patients due to the absence of an available blood sample decreased the statistical power and may have introduced bias. We postulate that the similarity among the data from the included and excluded patients with regard to the variables analyzed limited this bias. GnRH testing was not performed in six boys and three girls because of the presence of normal complete pubertal development and the high cost of GnRH testing. In girls, the inhibin B concentration was not measured in follicular phase when it is higher than in the luteal phase. In a significant number of patients, the concentration of inhibin B was measured many years after the serum samples were collected. However, the preservation of these samples in several aliquots at low and continuously controlled temperatures suggests that the ability to measure inhibin B was maintained. Furthermore, we found the same distribution as the normal concentrations in the five boys we previously reported [see participants in (9)]. The lack of genetic analysis in discriminating patients is also a limitation.

## Conclusions

This study shows the heterogeneity of pubertal abnormalities in patients with PSIS. Micropenis and cryptorchidism in boys and under-range serum inhibin B concentrations patients of both sexes might be suggestive of HH but may be absent or normal. As already reported in the literature, the serum inhibin B concentration is not a definitive for the diagnosis of HH, but it provides a simple first-line predictive test, especially in boys. The determination of the genes implicated in PSIS in the future may help in the understanding of the heterogeneity in the clinical-biological expression of this population.

## Data Availability Statement

The data that support the findings of this study are available from the corresponding author upon reasonable request.

## Ethics Statement

The studies involving human participants were reviewed and approved by The Comité de protection des personnes Ile de France III approved our studies on PSIS patients. Written informed consent from the participants' legal guardian/next of kin was not required to participate in this study in accordance with the national legislation and the institutional requirements.

## Consent for Publication

Informed consent was obtained from the individuals and minors' legal guardian/next or kin for the publication of any potentially identifiable images or data included in this article.

## Author Contributions

VC collected and interpreted the data, conducted data cleaning and statistical analysis, performed literature reviews, drafted and edited the manuscript. PL performed statistical analysis. SB-T supervised laboratory testing. RB conceived the study, oversaw the design and study coordination, carried out participant recruitment, drafted, and edited the manuscript. All authors read and approved the final manuscript.

## Conflict of Interest

The authors declare that the research was conducted in the absence of any commercial or financial relationships that could be construed as a potential conflict of interest.

## Abbreviations

ACTH: Adrenocorticotropic hormone
AUC: Area under the curve
CV: coefficient of variation
FSH: Follicle-stimulating hormone
GnRH: Gonadotropin-releasing hormone
GH: Growth hormone
HH: Hypogonadotropic hypogonadism
IGF1: Insulin-like growth factor 1
LH: Luteinizing hormone
MRI: Magnetic resonance imaging
PSIS: Pituitary stalk interruption syndrome
ROC: Receiver operating characteristic
TSH: Thyroid-stimulating hormone.
